# Are breast milk and serum irisin levels affected by the BMI and nutritional status? A prospective observational study

**DOI:** 10.1007/s00431-025-06154-0

**Published:** 2025-05-05

**Authors:** Feray Çağiran Yilmaz, Semra Türkoğlu, Fazilet Erman

**Affiliations:** 1 Department of Nutrition and Dietetics, Faculty of Health Sciences, Dicle University, Diyarbakır, Turkey; 2 Department of Nutrition and Dietetics, Faculty of Health Sciences, Firat University, Elazig, Turkey; 3 Department of Dietetic, Faculty of Health Sciences, Dicle University, Diyarbakır, Turkey

**Keywords:** Irisin, Obesity, BMI, Mothers, Infants

## Abstract

The influence of maternal nutritional status and anthropometric measurements on breast milk and serum irisin levels remains unclear. This study is the first to explore this relationship. This study aims to investigate the association between maternal BMI and nutritional status in the first month postpartum and their impact on breast milk and serum irisin levels. Forty-five mothers and their infants participated. Anthropometric measurements were taken at one month postpartum, maternal dietary intake was recorded over three days, and breast milk and serum irisin levels were analyzed. Overweight and obese mothers had lower breast milk irisin levels but higher serum irisin levels. A positive correlation was observed between breast milk irisin levels and infant birth weight. Additionally, serum irisin levels were positively associated with infant weight and height at one month. Maternal fiber intake was positively correlated with breast milk irisin levels, whereas fat intake showed a negative correlation. Moreover, higher folate, B12, and zinc intake were linked to increased breast milk and serum irisin levels.

*Conclusion*: Maternal BMI and nutritional status significantly influence breast milk and serum irisin levels. Promoting healthy eating habits and maintaining an optimal body weight before and after pregnancy may enhance irisin levels, potentially supporting infant growth and metabolic health.

**Table:** 

**What is known:**	
• *Irisin is a myokine involved in energy metabolism and insulin sensitivity, and breast milk composition is influenced by maternal BMI and nutritional status.* • *The effect of maternal obesity on breast milk irisin levels remains insufficiently understood.*	
**What is new:**	
• *This is the first study to evaluate the association between maternal BMI, nutritional status, and both breast milk and serum irisin levels.* • *Overweight and obese mothers exhibit lower breast milk irisin but higher serum irisin levels, and maternal intake of fiber, fat, folate, vitamin B12, and zinc is significantly associated with irisin levels.*

• *Irisin is a myokine involved in energy metabolism and insulin sensitivity, and breast milk composition is influenced by maternal BMI and nutritional status.*

• *The effect of maternal obesity on breast milk irisin levels remains insufficiently understood.*

• *This is the first study to evaluate the association between maternal BMI, nutritional status, and both breast milk and serum irisin levels.*

• *Overweight and obese mothers exhibit lower breast milk irisin but higher serum irisin levels, and maternal intake of fiber, fat, folate, vitamin B12, and zinc is significantly associated with irisin levels.*

## Introduction

After the discovery that adipose tissue has other functions, such as controlling various metabolic and immune processes including endocrine functions, it was recognized that excessive accumulation of adipose tissue was one of the main causes of metabolic and cardiovascular diseases. Basically, three different adipose tissues are emphasized. The white adipocyte represents the main energy reserve while the brown adipocyte is responsible for the oxidation of the lipids for thermogenesis. Beige adipocytes originate from white adipocytes and are involved in lipolysis and thermogenesis [1, 2].

Irisin, which was first discovered by Boström et al. in 2012 [3], is a myokine secreted into serum by skeletal muscles after exercise [3, 4]. Brown adipose tissue, which is abundant in infants and children, is in small amounts in adults [5]. Myokine is secreted in the skeletal muscle and liver, and its level increases with exercise [3]. Irisin, which is a recently identified hormone, promotes the browning of white and beige adipose tissues. This both increases thermogenesis and insulin sensitivity. It also helps lose weight by reducing glucose tolerance and fat mass [6, 7]. Another interesting aspect of this hormone is that it represents the link between muscle activity and lipolysis. Based on all these effects, it is thought that irisin plays a very important role in preventing obesity.

Breast milk is a dynamic and biological fluid that can be affected by many factors and it differs from mother to mother. Human breast milk contains 87–90% water and plays an important role as the primary source of water for newborns [8]. Breast milk contains cytokines, cells, steroids, bioactive substances, steroids, and enzymes. All these components found in breast milk play an important role in protecting the infant’s health [9]. Following the discovery of leptin [10] and ghrelin [11] in breast milk, the existence of other proteins was investigated. Firstly, it was determined that irisin was present in breast milk in the study of Aydın et al. [12]. Various studies supported that adipokines in breast milk played a role in many biological processes such as energy balance, appetite, glucose, and fat metabolism [13–15].

The current literature provides limited information on the influence of maternal nutritional status and anthropometric measurements on breast milk and serum irisin levels. Previous studies have confirmed the presence of adipokines such as leptin and adiponectin in breast milk and their potential effects on infant metabolic development. However, data on irisin levels in breast milk, their association with maternal factors, and their impact on infant growth and metabolic health remain scarce. This study aims to fill this critical gap by investigating the relationship between maternal BMI, nutritional status, and breast milk-serum irisin levels. The findings may contribute to a better understanding of the potential role of irisin in both maternal health and neonatal growth.

## Materials and methods

### Study design and data collection

This prospective observational study was conducted between January 15 and August 15, 2020, at Private Genesis Hospital in Diyarbakır, Turkey. Mothers were informed about the study during the last period of their pregnancy. The inclusion criteria included mothers who voluntarily agreed to participate, had given birth at term (≥ 37 weeks of gestation), had infants with a birth weight of ≥ 2.5 kg, and continued breastfeeding. Mothers diagnosed with gestational diabetes, preeclampsia, or other metabolic disorders, those using medication, smoking, or consuming alcohol, mothers who did not breastfeed, and those with preterm infants were excluded from the study. Mothers were contacted during the last trimester of pregnancy, and appointments were scheduled for those who voluntarily agreed to participate. After delivery, mothers were asked to visit the hospital with their infants between postpartum days 20 and 30 after fasting for at least eight hours. Demographic data, anthropometric measurements, and nutritional assessments were collected by an expert dietitian, while maternal blood samples were taken by an experienced hospital nurse. Blood and serum samples were analyzed by researchers with laboratory experience. All data were collected between postpartum days 20 and 30. Since mothers had different delivery dates, each mother was asked to visit the hospital within this time frame for data collection. The sample size of this study was determined based on a priori power analysis to ensure adequate statistical power. A power analysis was conducted using G*Power software, considering an effect size of *d* = 0.5 (moderate effect), an alpha level of 0.05, and a statistical power of 0.80 (80%). Given these parameters, the minimum required sample size for detecting significant differences was approximately 44 participants. To account for potential dropouts or missing data, 45 mothers and their infants were included in the study. No missing data were encountered during the study. All participants completed the required assessments. Before participation, mothers signed an informed consent form. Ethical approval was obtained from the Non-Interventional Research Ethics Committee of Fırat University on 17.09.2019 (approval number: 13/14).

### Anthropometric measurements

First month after birth, anthropometric measurements of the mothers were taken by the expert dietitian. The body weights of the mothers were measured in the mornings on empty stomach and without shoes while wearing light clothes. The measurements were conducted via an electronic scale with a 0.1-kg sensitivity while the heights were measured without shoes via a wall-mounted stadiometer with a 0.1-cm sensitivity. According to the standards of the World Health Organization (WHO), the mothers’ body mass indexes (BMI) were evaluated. Mothers with BMI values between 18.5 and 24.9 kg/m^2^ were classified as normal while those who were between 25.0 and 29.9 kg/m^2^ were classified as overweight in addition to those with ≥ 30.0 kg/m^2^ who were classified as obese [16]. Waist circumferences of the mothers were measured by considering the circumference in the midpoint passing through the interval between the lowest rib and the iliac crest while the circumferences of hip measured by from the highest part of the hips while mothers are on their side. Additionally, the middle upper arm circumferences were found by measuring the middle part of the ridge between the acromial and olecranon [17].

Body weight, height, and head circumferences of the infants were recorded according to gestational age. Infants’ body weights were measured with no clothes with a baby scale with a sensitivity of 10 g while their heights were measured with an infantometer in the lying position. Additionally, head circumferences were measured with a non-stretchable tape measure just above the eyebrows. Chest circumferences were measured by wrapping a tape measure over the widest part of the chest while the infant was in the supine position [18].

### Storage, collection, and analysis of samples

The samples of breast milk were collected between the 20 th and 30 th days postpartum (first month, mature milk) to ensure consistency in sampling during this stage. The 20 th to 30 th postpartum day was selected for sample collection to represent the mature milk stage and to minimize hormonal fluctuations that could affect irisin levels. To minimize variability due to circadian fluctuations, all samples were collected in the morning hours (between 08:00 and 10:00 AM). Mothers were instructed to breastfeed their infants before coming to the hospital, ensuring a minimum of two hours of breastfeeding interruption before sample collection. Then, 10 mL of breast milk was taken from one breast using an electronic breast pump (Mamajoo Inc., Germany-Turkey) without differentiating between foremilk and hindmilk to obtain a representative sample of the overall milk composition. The collected breast milk samples were immediately transferred into sterile, light-protected plastic containers and transported to the laboratory on ice. To prevent degradation and maintain biochemical integrity, the samples were stored frozen at − 80 °C. Before analysis, the samples were thawed under controlled conditions in a refrigerator at 4–6 °C. Furthermore, 3–4 mL blood samples were taken from the mothers to determine the serum irisin levels by transferring the blood samples into standard biochemistry tubes and separating the samples via centrifugation at 3000 rpm for 5 min at room temperature. Breast milk and serum irisin levels were measured with Human Irisin ELISA Kit (Catalog No: E3253HU).

### Food consumption status

During the preliminary interview with mothers in the last trimester of their pregnancy, the mothers who considered participating in the study were provided with training on keeping food consumption records. The participants were explained how to fill in the three-day food consumption registration form, portion sizes, and the importance of recording the snacks. The mothers were warned not to change their eating habits while recording their food consumption. Moreover, the mothers were asked to take their food consumption records within two consecutive days during the weekdays and one day on the weekends while recording the amount of food and the food consumed via household measurements. The book *Photo Catalog of FoodsandNutrients: MeasuresandAmounts* was used to determine the portions and amounts of the consumed foods [19]. The amount of nutrients included in the meals that the mothers consumed outside or whose contents the mothers did not know were determined in terms of quantity and size, and the book *Standard Recipes* was used to determine the amount of food in one portion consumed outside [20]. The mean energy and nutrient values of the foods consumed were calculated using the “Nutritional Information SystemsPackage (BEBIS)” program [21].

### Statistical evaluation of the data

In the evaluation of the statistical data, the Statistical Package for the Social Sciences (SPSS) package program was used. Shapiro Wilk and Kolmogorov Smirnov tests were used to determine whether the quantitative data were normally distributed while Student’s *t*-test was used in the evaluation of two normally distributed means. The Pearson correlation test was used to explore the strength and direction of the linear relationship between two normally distributed continuous variables. In the statistical tests, the confidence interval was regarded as 95%.

## Results

Anthropometric measurements of the mothers and the infants are presented in Table 1. It was determined that the mean age of the women participating in the study was 21.0 ± 5.7 years while their pre-pregnancy BMI values were 27.7 ± 4.1 kg/m^2^. Furthermore, their weight gains during pregnancy were 13.2 ± 4.6 kg. It was determined that the mean birth weight of the infants was 3.7 ± 0.5 kg while the height was 50.9 ± 1.2 cm. Additionally, the mean week of birth was 38.4 ± 1.2. Accordingly, it was determined that the mean term and ponderal index values of all the infants were 2.8 ± 0.4.

**Table 1:** Anthropometric data of the mothers and infants

Mothers’ and infants’ anthropometric data	Mean ± SD	Rank (min–max)
Maternal age (years)	21.0 ± 5.7	21.0–41.0
Pre-gestational weight (kg)	71.9 ± 9.5	53.0–94.0
Pre-pregnancy BMI (kg/m^2^)	27.7 ± 4.1	19.9–38.6
Weight gained during pregnancy (kg)	13.2 ± 4.6	6.0–25.0
BMI at end of gestation (kg/m^2^)	30.4 ± 3.2	26.6–40.3
Gestational age at birth (weeks)	38.4 ± 1.2	35.0–40.0
Weight at birth (kg)	3.7 ± 0.5	2.8–4.8
Height at birth (cm)	50.9 ± 1.2	47.0–53.0
Neonatal head circumference (cm)	34.2 ± 1.3	31.0–37.0
Neonatal thoracic perimeter (cm)	32.9 ± 0.9	31.0–35.0
Neonatal ponderal index (g/cm^3^ × 100)	2.8 ± 0.4	2.1–4.1

**Fig. 1:**
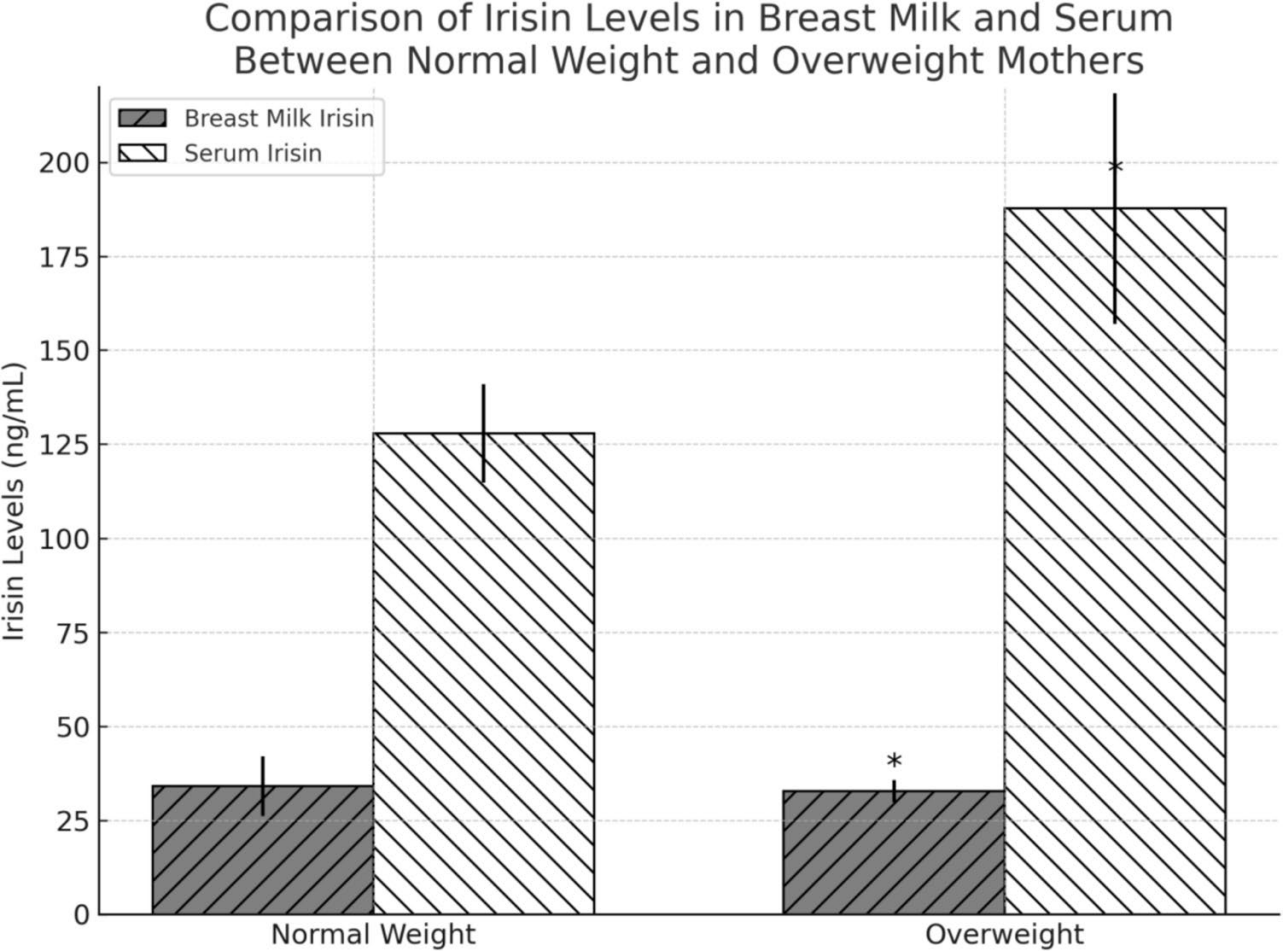
Comparison of maternal breast milk and serum irisin levels according to BMI. Breast milk and serum irisin levels (ng/mL) of normal weight (BMI 18.5–24.9 kg/m^2^) and overweight (BMI ≥ 25 kg/m^2^) mothers. Data are presented as mean ± SD. Statistical comparisons were made using Student’s *t*-test. *p < 0.05 indicates significant differences between groups

In Fig. 1, breast milk and serum irisin levels were presented based on BMI. While the irisin levels in the breast milk of the mothers who were overweight and obese were determined to be statistically significantly lower (breast milk irisin level of overweight mothers was 32.8 ± 2.9 ng/mL while breast milk irisin level of normal weight mothers was 34.1 ± 7.9 ng/mL, *p* = 0.035). Furthermore, serum irisin levels were determined to be high (serum irisin level of overweight mothers was 187.7 ± 30.7 ng/mL while breast milk irisin level of normal weight mothers was 127.9 ± 13.1 ng/mL, *p* = 0.035). In all the mothers, breast milk irisin level was determined to be lower than serum irisin level (*p* < 0.001).

The relationship between certain anthropometric values of the mothers and infants and breast milk-serum irisin levels is presented in Table 2. It was determined that there was a negative relationship between the BMI values of the mothers in the 1 st month after pregnancy and the breast milk irisin levels while there was a positive relationship between the serum irisin level, which were statistically significant (*p* values of 0.039 and 0.041, respectively). A positive correlation was observed between the body weights of the infants at birth and the first month and the irisin level in breast milk (*p* < 0.05). It was determined that there was a positive correlation between the body weights and heights of the infants in the 1^st^month after birth and serum irisin level (*p* = 0.033 and *p* < 0.001, respectively).

**Table 2:** The relationship between certain anthropometric data of mothers and infants and breast milk-serum irisin levels

Anthropometric data of mothers and infants	Irisin levels in breast milk		Serum irisin levels	
	***r***	p*	***r***	p*
Age	− 0.101	0.511	0.231	0.127
Mothers’ weight (kg)				
Pre-pregnancy	–0.150	0.327	0.265	0.078
1 st month after the pregnancy	–0.250	0.098	0.262	0.082
Mothers’ height (cm)				
Pre and post-pregnancy	–0.202	0.182	–0.073	0.634
Mothers’ BMI (kg/m^2^)				
Pre-pregnancy	–0.045	0.759	0.261	0.083
1 st month after the pregnancy	–0.133	**0.039**	0.306	**0.041**
Infants’ body weight (kg)				
Birth	0.288	**0.045**	0.144	0.344
1 st month after the birth	0.295	**0.038**	0318	**0.033**
Infant’s height (cm)				
Birth	–0.022	0.988	0.207	0.172
1 st month after the birth	–0.012	0.938	0.770	** < 0.000**
Infants’ head circumference (cm)				
Birth	–0.017	0.911	–0.119	0.436
1 st month after the birth	0.005	0.974	0.013	0.934
Infants’ chest circumference (cm)				
Birth	0.005	0.974	0.013	0.934
1 st month after the birth	–0.103	0.501	–0.251	0.096

^*^Pearson’s correlation test

The relationship between maternal macronutrient consumption and breast milk-serum irisin levels is presented in Table 3. It was determined that there was a negative relationship between the fat consumption of the mothers and the irisin level in breast milk, which was statistically significant (*p* < 0.05). A positive and significant relationship was discovered between the fiber intake of the mothers and the irisin level in breast milk (*p* = 0.001).

**Table 3:** The relationship between macronutrient intake of mothers and breast milk and serum irisin levels

Macronutrients	Breast milk irisin level		Serum irisin level	
	*r*	p*	*r*	p*
Energy (kcal)	–0.106	0.487	0.266	0.077
Carbohydrate (g)	0.151	0.322	0.093	0.545
Carbohydrate (%)	0.239	0.114	–0.071	0.645
Protein (g)	–0.017	0.911	0.078	0.611
Protein (%)	0.056	0.716	–0.099	0.518
Fat (g)	–0.291	**0.045**	0.208	0.171
Fat (%)	–0.307	**0.040**	0.095	0.535
Saturated fatty acids (g)	–0.230	0.128	0.013	0.934
Polyunsaturated fatty acids (g)	–0.231	0.127	0.258	0.087
Monounsaturated fatty acids (g)	–0.204	0.180	0.199	0.191
w-3 (g)	–0.008	0.959	–0.018	0.907
w-6 (g)	–0.085	0.579	–0.011	0.942
Fiber (g)	0.468	**0.001**	0.160	0.295
Soluble fiber (g)	0.414	**0.005**	0.188	0.217
Insoluble fiber (g)	–0.023	0.880	0.100	0.515

^*^Pearson’s correlation test

**Fig. 2:**
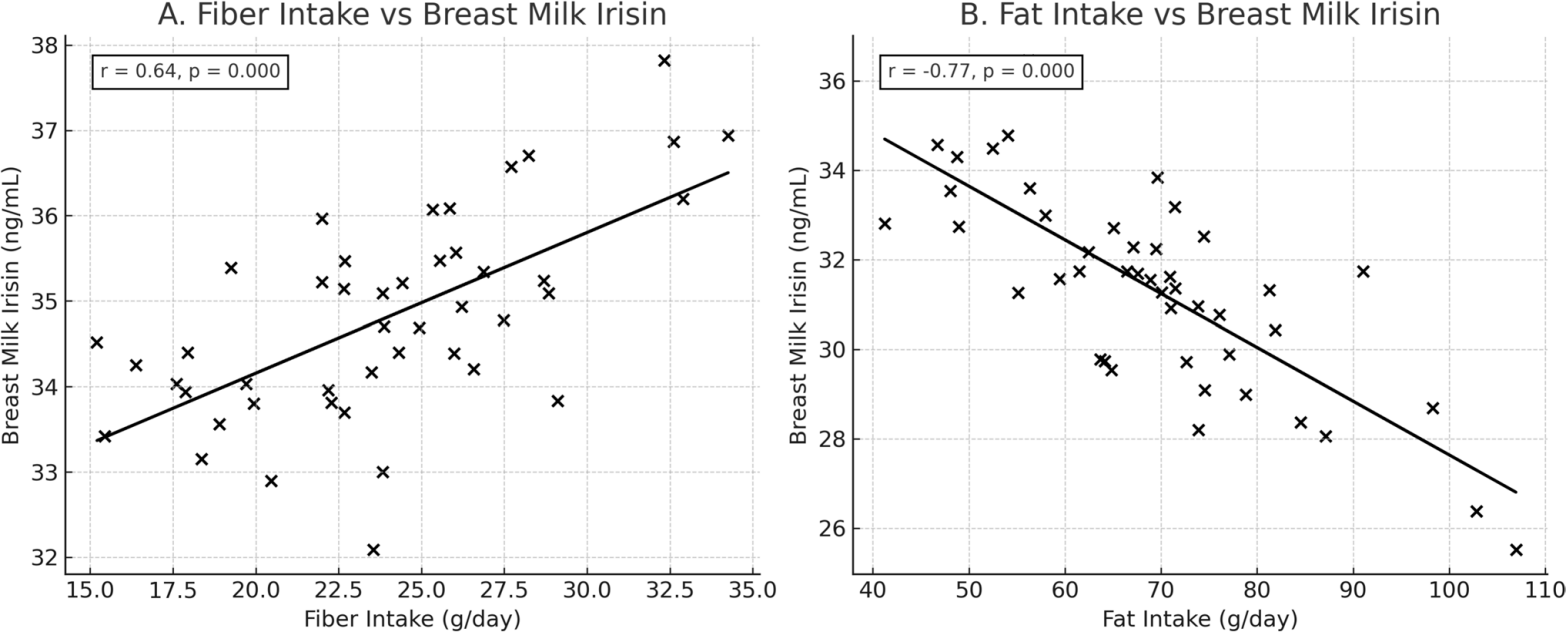
Correlations between maternal nutrient intake and breast milk irisin levels. **A** A significant positive correlation was observed between maternal fiber intake and breast milk irisin levels (*r* = 0.64, *p* = 0.000). **B** A significant negative correlation was observed between maternal fat intake and breast milk irisin levels (*r* = − 0.77, *p* = 0.000). Each point represents an individual participant. The solid lines represent linear regression trends

As shown in Fig. 2, a significant relationship was found between maternal macronutrient intake and breast milk irisin levels. A strong positive correlation was observed between daily fiber intake and breast milk irisin concentrations (*r* = 0.64, *p* = 0.000), indicating that higher dietary fiber may enhance the secretion of irisin into breast milk. Conversely, a strong negative correlation was detected between maternal fat intake and breast milk irisin levels (*r* = − 0.77, *p* = 0.000), suggesting that increased dietary fat may suppress irisin levels in breast milk. These findings highlight the potential influence of maternal dietary patterns on the hormonal composition of breast milk.

In Table 4, the relationship between mothers’ consumption of micronutrients and breastmilk-serum irisin levels was presented. It was determined that there was a positive relationship between folate, B12, and zinc intakes of mothers and both breast milk and serum irisin levels, which was statistically significant (*p* < 0.05).

**Table 4:** The relationship between mothers’ intake of micronutrients and breast milk and serum irisin levels

Micronutrients	Breast milk irisin level		Serum irisin level	
	*r*	p*	*r*	p*
Vitamin A (µg)	–0.051	0.741	0.077	0.616
Vitamin E (mg)	0.032	0.833	0.229	0.131
Vitamin B_1_ (mg)	0.113	0.458	0.165	0.279
Vitamin B_2_ (mg)	0.076	0.619	0.093	0.543
Niacin (mg)	0.277	0.066	0.224	0.139
Vitamin B_6_ (mg)	0.269	0.074	0.209	0.168
Folate (µg)	0.329	**0.027**	0.312	**0.037**
Vitamin B_12 (µg)_	0.421	**0.004**	0.326	**0.021**
Vitamin C (mg)	–0.248	0.100	0.159	0.295
Potassium (mg)	0.038	0.804	0.190	0.212
Calcium (mg)	0.138	0.367	0.117	0.445
Iron (mg)	–0.085	0.579	–0.011	0.942
Zinc (mg)	0.306	**0.041**	0.226	**0.038**

^*^Pearson’s correlation test

## Discussion

Irisin, which is also known as a myokine, is an adipokine that increases energy expenditure by stimulating the expression of uncoupling protein 1 (UCP1); thus, it promotes the browning of white adipose tissue [22, 23]. In addition to the beige or brownish fat cells, which are known to play a role in thermogenesis and energy expenditure in infants, brown fat itself was also identified in adults [24, 25]. Studies reported that the irisin serum levels increased in response to different types of exercises. Accordingly, it was suggested that irisin mediated certain health-promoting effects of physical activities [23, 26, 27]. Based on these findings, irisin has attracted considerable attention for its potential as an exercise hormone and in the future treatment of obesity [28–30].

Boström et al., in their pioneering study, detected irisin in serum from eight healthy human subjects [3]. Other studies extended these data and found that serum irisin levels were correlated with BMI while irisin correlated positively with body weight and BMI [31, 32].

Breastfeeding is predicted to protect against future obesity due to bioactive compounds found in breast milk, which are not found in infant formulas [33–35]. However, the specific components of breast milk, which are responsible for these beneficial effects, have not yet been identified clearly. Leptin, adiponectin, and irisin are proteins known to play a role in energy homeostasis and are present in breast milk. It is predicted that the level of irisin in breast milk contributes to the growth of infants and may be related to postnatal adaptation in terms of thermoregulation, vascular adaptation, glucose metabolism, lung function, and fluid homeostasis [36, 37]. It is also thought that the levels of these adipokines or myokines in milk may be affected by the mother’s condition and/or her nutrition during lactation. For example, breastmilk leptin and adiponectin levels of obese mothers were found to be higher than those of normal weight mothers [38–40]. In contrast, lactating women with gestational diabetes had lower irisin concentrations in breast milk compared to healthy lactating women [12]. In our study, the breast milk irisin levels of overweight and obese mothers were low (overweight mothers’ breast milk irisin level was 32.8 ± 2.9 ng/mL while normal weight mothers’ breast milk irisin level was 34.1 ± 7.9 ng/mL, *p* = 0.035) while the serum irisin levels were found to be high (overweight mothers’ serum irisin level was 187.7 ± 30.7 ng/mL while normal weight mothers’ breast milk irisin level was 127.9 ± 13.1 ng/mL, *p* = 0.035). Breast milk irisin levels were found to be lower than serum irisin levels in all mothers (*p* < 0.001). Furthermore, in our study, a positive correlation was discovered between breast milk irisin levels and infants’ birth weights and between serum irisin levels and weight and height values in the first month after birth. Low irisin level is regarded as a stress factor starting from the fetus and continuing until infancy. Irisin was proven to play a vital role in improving obesity and glucose homeostasis. Moreover, irisin was claimed to increase protein kinase phosphorylation, which is activated with adenosine monophosphate (AMP) and glucose intake; therefore, it plays an important role in glucose metabolism [9, 41]. Studies also supported the suggestion that irisin could inhibit atherosclerosis via endothelial proliferation and cholesterol synthesis in hepatocytes in addition to providing osteoblast proliferation and differentiation. Based on all these effects of irisin, it was thought that low irisin levels in breast milk during lactation could cause adverse effects on infants’ health and glucose metabolism [42–44].

In the literature, in rats that were subjected to the “cafeteria diet,” which is a diet similar to a Western-style diet with delicious snacks offered in addition to a balanced diet, it was determined that total energy and fat intakes were increased while protein intake was decreased. It was also determined that these changes in the macronutrient composition of the maternal diet were reflected in the composition of breast milk [35, 45]. There is not enough information about breast milk irisin levels and the role of irisin-its relationship with maternal nutrition. In another study, it was determined that the cafeteria diet conducted with rats decreased the milk irisin levels of the rats; however, there was no relationship between the milk irisin level and maternal adiposity [35]. Similarly, in our study, it was determined that there was a negative relationship between mothers’ fat consumption and breast milk irisin level, which was statistically significant (*p* < 0.05). A positive and significant relationship was discovered between the fiber intake of the mothers and the irisin level in breast milk (*p* = 0.001). Furthermore, it was determined that there was a positive relationship between mothers’ folate, B12, and zinc intakes and both breast milk-serum irisin levels, which was statistically significant (*p* < 0.05).

Previous studies have demonstrated that various bioactive hormones in breast milk, such as leptin and adiponectin, play crucial roles in infant metabolism, energy homeostasis, and growth. Research has shown that leptin levels in breast milk are positively correlated with maternal BMI, with overweight and obese mothers having higher leptin concentrations compared to normal-weight mothers. Similarly, adiponectin has been found to be influenced by maternal metabolic status, with lower levels reported in obese mothers [10–38]. In contrast, our study found that breast milk irisin levels were lower in overweight and obese mothers, while serum irisin levels were higher in this group. This pattern differs from leptin and adiponectin, suggesting that irisin may be regulated differently in maternal metabolism. Additionally, our findings revealed a positive correlation between breast milk irisin levels and infant birth weight, similar to some studies on leptin, which has been associated with infant growth and appetite regulation [39].

This study has some limitations. Although this study is prospective, further studies with larger sample sizes and longer follow-up periods should be conducted. Additionally, examining hormones other than irisin will further contribute to the literature. Potential confounding variables such as physical activity, type of feeding, and metabolic conditions were not adjusted for in the statistical analyses. This represents a limitation of the current study. While this study presents valuable findings, the generalizability of the results is limited due to the small sample size. Therefore, future studies with larger sample sizes will be essential to support these findings.

In conclusion, this study is the first to examine the relationship between maternal nutritional status, anthropometric measurements of mothers and infants, and breast milk-serum irisin levels and it yielded statistically significant and biologically relevant findings. Based on the results of this study, it was determined that both breast milk and serum irisin levels were associated with the mothers’ BMI and nutritional status. Having a healthy body weight and adopting a healthy diet can positively affect the level of irisin in breast milk; therefore, it is rather important for mothers to receive the necessary training in this regard.

## Data Availability

No datasets were generated or analysed during the current study.
